# Development of a novel recombinant ELISA for the detection of Crimean-Congo hemorrhagic fever virus IgG antibodies

**DOI:** 10.1038/s41598-021-85323-1

**Published:** 2021-03-15

**Authors:** Sultan Gülce-İz, Nazif Elaldı, Hüseyin Can, Esra Atalay Şahar, Muhammet Karakavuk, Aytül Gül, Gizem Örs Kumoğlu, Aysu Değirmenci Döşkaya, Adnan Yüksel Gürüz, Aykut Özdarendeli, Philip Louis Felgner, Huw Davies, Mert Döşkaya

**Affiliations:** 1 Department of Bioengineering, Faculty of Engineering, Ege University, Izmir, Turkey; 2 Department of Physiology and Biophysics, Vaccine Research and Development Center, University of California, Irvine, CA USA; 3 Department of Infectious Diseases and Clinical Microbiology, Sivas Cumhuriyet University, Faculty of Medicine, Sivas, Turkey; 4 Department of Biology, Section of Molecular Biology, Ege University, Faculty of Science, Izmir, Turkey; 5 Department of Biotechnology, Ege University, Faculty of Engineering, Izmir, Turkey; 6 Department of Parasitology, Ege University, Faculty of Medicine, Izmir, Turkey; 7 Blood Bank of Ege University, Ege University, Faculty of Medicine, Izmir, Turkey; 8 Department of Medical Microbiology, Erciyes University, Faculty of Medicine, Kayseri, Turkey

**Keywords:** Viral infection, Diagnostic markers, Molecular medicine

## Abstract

Crimean-Congo hemorrhagic fever (CCHF) is a tick-borne viral infection caused by Crimean-Congo hemorrhagic fever virus (CCHFV). Serological screening of CCHF is important and current ELISA use antigens prepared from virus which is expensive due to requirement of high bio-containment facilities. In this study, we aimed to develop a new recombinant ELISA. For this purpose, CCHFV genome were expressed as 13 proteins in *E. coli* and among them abundantly purified recombinant Nucleocapsid protein (rNP) and Mucin-like variable domain (rMLD) were used as antigen in ELISA (Rec-ELISA). Rec-ELISA using rNP, rMLD and a combination of both (rNP/rMLD) were probed with acute (n = 64; collected between days 1 and 7 after onset of symptoms), convalescent (n = 35; collected 8 days after onset of symptoms), consecutive sera (n = 25) of confirmed CCHF cases and control sera (n = 43). The sensitivity and specificity of Rec-ELISA using rNP/rMLD were 73% and 98% in acute cases and 97% and 98% in convalescent cases. The median interquartile absorbance value to discriminate the acute and convalescent phases of CCHF was significantly higher with ELISA using rNP/rMLD (*P* < 0.0001) compared to rNP (*P* > 0.05) and rMLD (*P* = 0.001). These results indicate that the Rec-ELISA using rNP/rMLD may be very useful to diagnose convalescent CCHF cases especially in field studies.

## Introduction

Crimean-Congo hemorrhagic fever (CCHF) is an acute, highly-contagious and life-threatening viral infection with a case fatality rate of up to 30%^1^. The causative agent, CCHF virus (CCHFV), is an orthonairovirus of the *Nairoviridae* family. The virus is transmitted to humans through a bite from an infected tick or direct contact with infected body fluids of patients. The ixodid tick *Hyalomma m. marginatum*, plays a major role for the transmission of the disease and it is the natural reservoir of CCHFV^1–4^. CCHF is a neglected disease and re-emerging due to the climate change effecting tick distribution worldwide^5^. This is supported by the occurrence of CCHF in more than 30 countries in parts of Africa, Asia, Eastern Europe, and the Middle East^2^.

Although the disease was first recognized in an outbreak in Crimea in 1944, it has been showed that the virus was identical to the one isolated from a patient in Congo in 1956. Thereafter, the name of the virus became “Crimean-Congo Hemorrhagic Fever”^6^. CCHFV circulates in Turkey for several decades and the first human cases were identified in Kelkit valley of Turkey in 2002^3,7–9^.

The CCHFV genome consists three negative polarity RNA segments which are called small (S), medium (M) and large (L) based on their genome sizes^10^. The S segment which encodes nucleocapsid protein (NP) is the major protein primarily detected during the viral invasion phase of CCHF^10–13^. NP expressed in bacteria^5,14–20^ and plants^21^ have been used to diagnose CCHF using ELISA. In addition, NP has immunodominant peptides that interact with IgG and IgM antibodies^13,22^. A DNA vaccine encoding full-length NP induced strong humoral and cellular immune responses indicating the high immunogenicity of NP^12^. The M segment encodes a glycoprotein precursor that is processed into two structural glycoproteins (Gn and Gc), a non-structural protein (NSm) found on the surface of virion^23^, and the secreted non-structural glycoproteins (NSGs) GP85, GP160, and GP38^24^. NSGs contain mucin-like domain (MLD) and a GP38 domain^25^. The L segment encodes a RNA-dependent RNA polymerase with a molecular weight of ~ 450 kDa that replicates and transcribes the viral RNA^26^.

Since the CCHFV is considered to be one of the major emerging infectious threats, rapid diagnosis in the field after an outbreak to guide the measures to be taken is very important^4^. Better diagnostic assays will improve efficiency of treatment in humans and reservoir animals.

The serological diagnosis of CCHF can be achieved by ELISA kits detecting *anti-*CCHFV specific antibodies. ELISAs frequently use antigen derived from the virus propagated in cell culture or brain tissue of suckling mice^21^. This is an important problem since CCHFV is a high level biosafety pathogen and thus the preparation of the antigen requires high bio-containment facilities^27^. World Health Organization’s “WHO R&D Blueprint: Priority Diagnostics for CCHF” documentation prioritized the need for development and validation of new serological tests including ELISA in 2019^28^. To address this necessity, ELISAs using recombinant NP protein of CCHFV are being developed^16,17,19,21,27,29^.

In this study, we aimed to develop an ELISA using more than one recombinant protein of CCHFV as antigen, to detect *anti-*CCHFV IgG antibodies in acute and convalescent phases of the illness. For this purpose, 13 segments of the CCHFV genome [S, M (divided into three), L (divided into nine)] were expressed in *E. coli* and purified as recombinant protein. The most abundantly expressed recombinant proteins were used as antigen in ELISA probed with sera of well-categorized CCHF patients from Turkey.

## Results

### Characteristics of patients and controls

In this study, 64 of 93 serum samples collected from CCHF suspected patients resulted with CCHFV positivity according to the reference tests of RT-PCR and/or IFA detecting anti-CCHFV IgM. Accordingly, the prevalence of CCHF was 68.8% (64/93) in this group of patients.

Among the 64 CCHF positive patients, 62 (96.9%) of them were confirmed by RT-PCR and the remaining two patients (3.1%) were confirmed by the anti-CCHFV IgM IFA test in convalescent phase serum samples. The remaining 29 (31.2%) RT-PCR and IFA negative samples were excluded from the study. A total of 43 healthy blood donors (29 male, 67.4%) were also included as the control group. The mean ± standard deviation (range) age for the 64 (40 male, 62.5%) acute phase CCHF patient group was 45.7 ± 15.3 (17–78) years and it was 41.3 ± 10.3 (19–60) years for the control group, respectively (*P* = 0.076). Acute phase CCHF patient and control groups were also found to be the identical for gender (*P* = 0.64).

The median (IQR) time of symptom duration before the date of CCHFV diagnostic testing (sampling day) for the 64 acute phase CCHF patients was 4 (3–6) days (range 1–7 days). The median (IQR) sampling time after onset of symptoms for the 35 convalescent phase CCHF patients was 10 (9–13) days (range 8–25 days). Furthermore, 25 CCHF patients had two sera samples collected consecutively in the acute (hospitalization day) and convalescent (discharge day) phases of the illness. Among the patients diagnosed with CCHF (n = 64), 62 (96.9%) of them recovered and two of them (3.1%) died.

### Expression, selection, and purification of recombinant proteins

**Figure 1:**
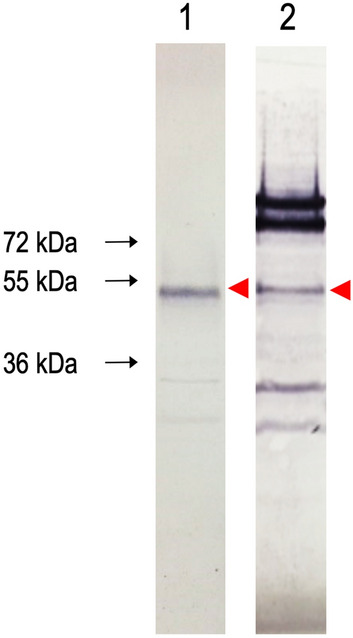
Western blotting images showing the purified rNP and rMLD proteins. Lane 1: rNP protein with a theoretical molecular weight of ~ 53.9 kDa; Lane 2: rMLD protein with a theoretical molecular weight of ~ 53.67 kDa. Western blot images are obtained from separate blots and delineated with white space. Arrowheads next to each blot show the place of purified recombinant proteins. Original Western blot images of Lane 1 and 2 are shown in Supplementary Figs. S3 and S4, respectively.

13 segments of the CCHFV genome isolated by PCR were inserted into pXT7 plasmids using in vivo homologous recombination cloning. After small scale expression of each recombinant CCHF protein in *E. coli*, the recombinant proteins were purified using Ni–NTA beads. Among the 13 recombinant proteins, rNP and rMLD with calculated molecular masses of ~ 53.8 kDa and ~ 53.6 kDa, respectively were expressed at acceptable levels for assay development (Protein expression levels of 13 recombinant CCHFV proteins as detected by Western blot using anti-polyhistidine antibody are shown in Supplementary Figs. S1, S2.). Then, *E. coli* cells expressing rNP and rMLD were grown in a bioreactor, purified to homogeneity using Ni + 2 chelating column followed by gel filtration (Fig. 1).

### Evaluation of IgG Rec-ELISA using acute and convalescent sera of CCHF patients

In this study, Rec-ELISAs using recombinant rNP, rMLD, and rNP/rMLD were developed to determine the *anti*-CCHFV IgG antibodies in sera collected from CCHF patients during the acute (day 1–7) and convalescent (day 8–25) phases of the illness. The sensitivity and specificity values of Rec-ELISAs detecting IgG antibodies in acute and convalescent sera are shown in Tables 1 and 2.

**Table 1:** Comparison of test results for the Rec-ELISA using rNP, rMLD, and rNP/rMLD probed with sera of acute phase CCHF patients (n: 64).

Antigens	Results	Groups		*P* value	Odds ratio (95% CI)
		Acute CCHF (n = 64)	Control (n = 43)		
rMLD	Positive	44 (68.8%)	1 (2.3%)	** < 0.0001**	92.4 (11.9–720.0)
	Negative	20 (31.2%)	42 (97.7%)		
rNP	Positive	46 (71.9%)	1 (2.3%)	** < 0.0001**	107.0 (13.7–840.0)
	Negative	18 (28.1%)	42 (97.7%)		
rNP/rMLD	Positive	47 (73.4%)	1 (2.3%)	** < 0.0001**	116.0 (14.8–911.0)
	Negative	17 (26.6%)	42 (97.7%)		

*CI* confidence interval.

Bold values are statistically significant for *P* <0.05.

**Table 2:** Comparison of test results for the Rec-ELISA using rNP, rMLD, and rNP/rMLD probed with sera of convalescent phase CCHF patients (n: 35).

Antigens	Results	Groups		*P* value	Odds ratio (95% CI)
		Convalescent CCHF (n = 35)	Control (n = 43)		
rMLD	Positive	31 (88.6%)	1 (2.3%)	** < 0.0001**	326.0 (34.6–3059.0)
	Negative	4 (11.4%)	42 (97.7%)		
rNP	Positive	34 (97.2%)	1 (2.3%)	** < 0.0001**	1428.0 (76.0 – 23,699)
	Negative	1 (2.8%)	42 (97.7%)		
rNP/rMLD	Positive	34 (97.2%)	1 (2.3%)	** < 0.0001**	1428.0 (76.0 – 23,699)
	Negative	1 (2.8%)	42 (97.7%)		

*CI* confidence interval.

Bold values are statistically significant for *P* <0.05.

The Rec-ELISA using rNP/rMLD antigen showed the highest sensitivity of 73% [95% confidence interval (CI) 61–84] in acute sera and a specificity of 98% (95% CI 89–100) in the control group sera (Table 1). The positive predictive value (PPV) of Rec-ELISA using rNP/rMLD antigen was 98% (95% CI 89–100) and the negative predictive value (NPV) was 71% (95% CI 58–82) (Table 3). Specifically, for the convalescent sera, Rec-ELISA using rNP/rMLD antigen had a sensitivity of 97% (95% CI 85–100) and a specificity of 98% (95% CI 88–100) (Table 2) with a PPV and NPV of 97% (95% CI 85–100) and 98% (95% CI 88–100), respectively (Table 3).

**Table 3:** The efficacy of Rec-ELISA using rNP, rMLD, and rNP/rMLD for the detection of *anti*-CCHFV IgG antibodies in sera of acute and convalescent CCHF patients

	Rec-ELISA using		
	rMLD	rNP	rNP/rMLD
**Acute CCHF (day 1–day 7)**			
Sensitivity	69% (56–79)	72% (59–82)	73% (61–84)
Specificity	98% (88–100)	98% (88–100)	98% (89–100)
Positive predictive value (PPV)	98% (88–100)	98% (89–100)	98% (89 -100)
Negative predictive value (NPV)	68% (55–79)	70% (57–81)	71% (58–82)
Likelihood ratio	29.6%	30.9%	31.6%
**Convalescent CCHF (day 8–25)**			
Sensitivity	89% (73–97)	97% (85–100)	97% (85–100)
Specificity	98% (88–100)	98% (88–100)	98% (88–100)
Positive predictive value (PPV)	97% (84–100)	97% (85–100)	97% (85–100)
Negative predictive value (NPV)	91% (79–98)	98% (88–100)	98% (88–100)
Likelihood ratio	38.1%	41.8%	41.8%

Except for Likelihood ratio, data presented as percent (95% confidence Interval).

**Figure 2:**
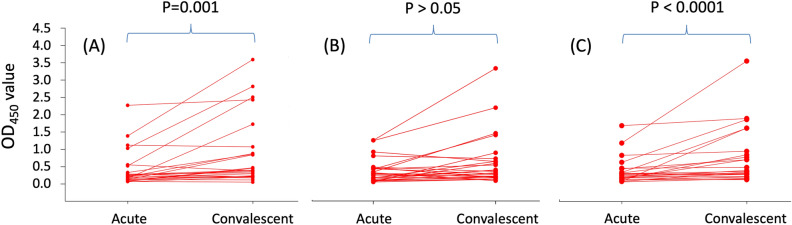
Differences in absorbance values obtained from Rec-ELISA using (**A**) rMLD, (**B**) rNP, and (**C**) rNP/rMLD probed with the consecutive sera collected from CCHF patients during acute (1–7 days) and convalescent (8–25 days) phase (n: 25). Acute *vs.* convalescent median (IQR) absorbance value [optical density (OD)] of Rec-ELISAs using (**A**) rMLD was 0.21 (0.13–0.43) *vs.* 0.39 (0.22–0.98) with a *P* = 0.001, (**B**) rNP, 0.34 (0.14–0.52) *vs.* 0.42 (0.24–0.77); *P* > 0.05, and (**C**) rNP/rMLD, 0.26 (0.15–0.42) *vs.* 0.39 (0.27–0.94); *P* < 0.0001. The results were evaluated in a micro titer plate reader (Bio-Tek ELx808, USA) at 450 nm and expressed as OD. Data were analyzed by Wilcoxon signed-rank test. Cut-off values of each plate ranged between 0.140 and 0.152 (OD_450_ value).

In the group of 25 CCHF patients who had two serum samples collected consecutively during the acute and convalescent phases, the median interquartile absorbance value (IQR) to discriminate the acute and convalescent phases of CCHF was significant with ELISA using rNP/rMLD [0.26 (0.15–0.42) *vs* 0.39 (0.27–0.94); *P* < 0.0001; effect size r = 0.61] (Fig. 2) and Rec-ELISA using rMLD [0.21 (0.13–0.43) *vs*. 0.39 (0.22–0.98) with a *P* = 0.001; effect size r = 0.59]. In Rec-ELISA using rNP, there were no significant difference between the acute and convalescent phase of serum samples [0.34 (0.14–0.52) *vs* 0.42 (0.24–0.77); *P* > 0.05; effect size r = 0.42] (Fig. 2).

In Fig. 2, OD_450_ values of most patients showed an increase in convalescent sera. In consecutive sera of two patients, OD_450_ values decreased in all assays. OD_450_ values decreased in two patients in Rec-ELISA using rNP and rMLD or rNP/rML and slightly decreased in one patient assayed with rMLD or rNP/rMLD, and four with rNP ELISA.

According to the Pearson correlation analyses, highest comparable results obtained by Rec-ELISAs using rMLD and rNP as well as rNP/rMLD and rNP were achieved in convalescent sera. Comparison of Rec-ELISAs using rMLD/rNP with rMLD showed that the highest comparable results were obtained with acute sera obtained from patient group with consecutive sera (Supplementary Fig. S5).

## Discussion

The CCHFV diagnosis can be achieved by RT-PCR, antigen-capture ELISA (cELISA), IFA, ELISA, immunohistochemical staining of infected tissues, or isolation of virus^29^. In human patients who survived from CCHF (IgG seroconversion does not occur in cases with fatal outcome), the IgG antibodies become detectable from the second week of the infection^30,31^. In animals, IgG seroconversion occurs after a short viremia of up to 2 weeks^28^. It is important to emphasize that the infection initiates as the virus is transferred to the body by tick bite or contact with contagious body fluids. Therefore, both in animal and human populations, seroprevalence studies using ELISA can reveal CCHF high risk areas and may help to determine the transmission potential of CCHF animals to humans.

Several commercial IFA and ELISA test kits, in the market for the detection of CCHF specific IgG antibodies, use the virus itself, cells expressing CCHFV glycoprotein precursor, recombinant NP and unknown target antigens as antigen^27,32^. Although the kits that utilize virus itself as antigen have a high sensitivity and specificity, their production is expensive since continuous production of CCHFV requires high level biosafety laboratory conditions. CCHFV is classified as a biosafety level 4 pathogen in countries where CCHF is not detected^33^.

Recently WHO prioritized the development and validation of new serological tests including ELISA for the field screening of CCHF^28^. Thus, ELISAs using the recombinant CCHFV protein as an antigen to detect the CCHF specific antibodies in human samples is required by the health authorities and market. To address this necessity, ELISAs using recombinant NP protein of CCHFV are being developed^16,17,19,21,27,29^. In a study conducted in South Africa, the researchers developed an indirect ELISA using recombinant NP expressed in *E. coli* to detect *anti*-CCHFV IgG antibodies in serum samples of 14 acute confirmed CCHF patients. Although the recombinant antigen was able to detect IgG antibody in acute and convalescent sera of recombinant NP immunized mice, it was unable to detect IgG antibody in human serum samples collected during the 15–16 days of the illness^16^. Two years later, the same researchers reported that the ELISA using different *E. coli* expressed recombinant NP antigens with genetic diversity from different strains showed different sensitivity rates in the sera collected during acute phase of illness^17^. In this study, they developed ELISA using recombinant NP antigens isolated both from a patient in South Africa and a tick from Greece with high genetic diversity and probed them with the serum samples of 14 confirmed human CCHF cases again. They stated that the ELISA kits using recombinant NP developed from a South African CCHFV strain detected IgG antibody in all (100%), but the other ELISA kit using recombinant NP developed from an AP92 Greek CCHFV strain detected 13 (92.9%) of 14 CCHF patients^17^. These results show that there is little evidence to support serological strain differences between heterologous antigen and sera antigenicity.

In a recent multicenter study, an ELISA kit using *E. coli* expressed recombinant NP of the IbAr10200 strain was probed with the serum samples of CCHFV infected ruminants (95 cattle and 176 small ruminants) from CCHF-endemic regions including Albania, Cameroon, Kosovo, Former Yugoslav Republic of Macedonia, Mauritania, Pakistan, and Turkey. The sensitivity and specificity for the detection of IgG antibody of ELISA was reported as 98.8% and 100%, respectively^18^. This study indicated that the origin of the sera may not have that much influence on the ELISA results regarding strain variation. In another parallel study conducted in animal samples, an ELISA test kit using recombinant codon optimized NP as antigens, detected *anti*-CCHFV IgG antibodies in 76 suspected samples with 79.4%sensitivity and 100% specificity^20^.

In a study, the NP specific monoclonal antibodies were generated against an *E. coli* expressed NP and used in a species-independent capture ELISA to evaluate 833 serum samples collected from animal and humans which showed 95% sensitivity and 99% specificity^5^. An indirect ELISA kit using a plant produced recombinant NP detected *anti-*CCHFV IgG antibodies in all of the 13 convalescent phase of CCHF serum samples and showed 100% sensitivity and specificity when the results compared with 13 control samples^21^. The evaluation of an *in house* IgG immune complex (IC) ELISA kit utilizing the recombinant NP for the detection of *anti*-CCHFV IgG antibodies in sera collected from 15 patients in acute stage and 12 follow up sera of the same patients collected 1 year after recovery revealed that all the sera collected between day 11 and day 19 days were IgG positive, whereas a commercial IgG ELISA kit detected only 27% of them. Both the *in house* and commercial kits detected high IgG titers in the convalescent patients sera collected 1 year after recovery^19^.

In the present study, a Rec-ELISA using a combination of recombinant protein of full length S-segment (rNP) and Mucin-like domain of M-segment (rMLD) have been tested for the first time in well characterized acute (onset of symptoms between 1 and 7 days) and convalescent (8–25 days) sera collected from confirmed CCHF patients in Turkey. The rNP and rMLD proteins were selected based on the expression and purification yield since recombinant ELISA requires abundant amount of purified protein as antigen. Nonetheless, *E. coli* expression system does not always yield the required amount of protein in each transformant. In addition, we aimed to use several proteins as antigen in ELISA however more antigens do not always improve the ELISA and specificity and sensitivity of the recombinant ELISA could be improved by adding the right combinations of antigenic proteins^34^. The 25 serum samples collected consecutively from the same CCHF patients during the acute and convalescent phases were also tested for the presence of *anti*-CCHFV IgG antibodies by recombinant Rec-ELISAs. In the group of acute patients, the sensitivity of Rec-ELISAs using rNP and rNP/rMLD were 72% and 73% respectively compared to Rec-ELISA using rMLD which had 69% sensitivity. In the group of convalescent patients, the highest sensitivity (97%) rate was achieved by Rec-ELISA using rNP/rMLD or rNP and the sensitivity rate of the ELISA using rMLD remained as 89%. The specificities were the 98% for all ELISAs.

Most importantly, in the group of 25 consecutive sera, the median interquartile absorbance value (IQR) to discriminate the acute and convalescent phases of CCHF was significant with ELISA using rNP/rMLD (*P* < 0.0001) and rMLD (*P* = 0.001) whereas for ELISA using rNP there were no significant change (*P* > 0.05). These results indicate that the Rec-ELISA using rMLD in combination with rNP can be useful to diagnose CCHF in field screenings as the absorbance values of ELISA using rMLD in combination with rNP is higher than ELISA using solely rNP as antigen (Fig. 2). Therefore, Rec-ELISAs developed in this study worked better in the convalescent sera compared to acute sera possibly due to occurrence of IgG antibodies between days of 5 and 9 days after the onset of symptoms^16,35,36^. In the control group, the OD value for a serum sample of one donor was found to be higher than the cut-off level which may be giving cross reactivity with a virus with similar antigenic properties with CCHFV or may be infected with CCHFV by exposure a tick in the past and was recovered by mild disease symptoms. A seroepidemiologic survey from Turkey reported 88% subclinical CCHF infection^37^. The cross reactivity of rNP and rMLD may also be analyzed with sera of patients with confirmed non-CCHFV infections to improve the research. However, studies showed that IgG antibody against Dugbe and Hazara viruses, related Nairoviruses and infectious for humans, did not cross-react with rNP in ELISA^38,39^.

In conclusion, the results of this study show that Rec-ELISA using a combination of recombinant full length Nucleocapsid and Mucin-like domain of CCHFV (rNP/rMLD) detects *anti*-CCHFV IgG antibodies with high sensitivity (97%) in the group of convalescent phase serum samples collected after day 8 of the illness and has 73% sensitivity in the group of acute phase serum samples. In addition, the discriminatory power of Rec-ELISA using two antigens is better compared to using one antigen. Therefore, it may be better to use more than one recombinant protein as antigen in future CCHF ELISA development studies and surveillance studies about CCHF.

## Methods

### Biosafety

In Turkey, clear procedures governed by institutional safety committees are in place to protect against CCHFV transmission in the hospital and laboratory setting. These procedures include the use of personal protective equipment (PPE) and equipment, as well as sample transfer. All researchers were trained in mitigating biohazards associated with CCHFV according to approved local IBC protocols. The BSL-3 laboratory located in the Department of Microbiology, Erciyes University (Kayseri, Turkey) was used to process CCHFV strain and inactivation of serum samples. Methodological details are provided below. Plasmids were constructed in the University of California, Irvine, and recombinant proteins were expressed and purified in BSL-2 facility at the Department of Parasitology, Ege University (Izmir, Turkey) in accordance with approved Institutional Biosafety Committee protocols. Recombinant protein ELISAs were performed at BSL-3 laboratory in the Department of Medical Microbiology, Erciyes University. All work plans undergo risk assessment and regular review by the Health & Safety Executive. All methods were performed in accordance with the relevant guidelines and regulations. Sample transfers between the centers and the laboratory (located in the same campus) were performed according to standard guidelines developed by the USA Centers for Disease Control and Prevention (CDC)^40^.

### Study design and patients and controls

CCHF-suspected patients who were hospitalized and followed-up at the Department of Infectious Disease and Clinical Microbiology at Faculty of Medicine, Sivas Cumhuriyet University (Sivas, Turkey) were enrolled to this study. The patients having at least two of the following findings: fever, headache, diffuse body pain, arthralgia, weakness, diarrhea and bleeding, in addition to visiting a CCHF endemic region within the last 2 weeks or residing in an endemic region or history of tick exposure with thrombocytopenia (150,000/mm^3^ and/or leukopenia (< 4000/mm^3^), were considered as suspected CCHF cases^41^. The diagnosis of CCHF was confirmed by real-time PCR and/or by the IFA anti-CCHFV IgM test in both the acute and convalescent cases. Sera were anonymized and retrospectively used during the study. Adult Turkish healthy blood donors who do not have any chronic disease including diabetes mellitus, chronic liver disease, chronic renal failure and malignity, and do not show any clinical symptoms related with CCHF and have no history of being bitten by tick previously, were accepted as the control group. Each patient’s and control subject’s age and gender, time to hospital (*d*) and discharge day after symptoms onset (the illness day) were recorded into a database. To discharge from hospital, patient should have no findings of hemorrhage in addition to normal coagulation values and increased platelet counts (≥ 100,000/mm^3^)^41^.

### Serum collection, CCHF diagnosis, and inclusion criteria

The first serum sample was obtained from all CCHF-suspected patients by venipuncture on the admission date and if it is possible, from the survivors, a second serum sample was obtained on the discharge date. The sera samples obtained from each CCHF suspected patient at admission were sent to the CCHF Reference Center, Ankara, Turkey for the detection of the CCHFV nucleic acid using a commercial real-time RT-PCR test (Altona Diagnostics, Hamburg, Germany). All RT-PCR negative acute phase sera samples were also tested by the commercial IgM indirect immunfluorescence antibody (IFA) assay (Euroimmun, Luebeck, Germany). If such samples were negative by the IFA IgM test, a second convalescent phase of serum samples collected discharge day were also sent to the reference laboratory to repeat IFA IgM test. Aliquots of remaining sera both from the acute and convalescent phase of serum samples were collected and stored at − 80 °C. Patients having a positive PCR in acute phase and/or IgM seropositivity in acute or convalescent phase serum samples were diagnosed as CCHF and included into the study. Serum samples for the CCHF patients were later classified as acute (1–7 days) or convalescent (day 8 and later) based on the onset of CCHF symptoms and retrospectively used during the study.

### Construction of plasmids

The total RNA of CCHFV was extracted from an acute phase serum sample of CCHF patient using RNeasy Kit (Qiagen, USA) and cDNA was obtained by Qiagen QuantiTect Reverse Transcription Kit (Qiagen, USA) according to the manufacturer’s protocol. All of the remaining reagents were purchased from the Applichem (Germany) unless otherwise stated. 13 segments of the CCHFV-Kelkit06genome [GenBank Accession No: GQ337053.1 for S segment, GQ337054.1 for M (divided into three), GQ337055.1 for L (divided into nine) segments] were amplified using appropriate primers (Supplementary Table S1) and transformed using high-throughput PCR and recombination cloning method as described previously^42,43^. During PCR, gene specific primers (Supplementary Table S1) containing 18–22 bp nucleotide extension complementary to ends of linearized pXT7 vector with kanamycin resistance were used (Supplementary Table S1). For S segment, 1448 bp ORF between 56 and 1503 nucleotides (482 aa), for M-mucin like segment 1489 bp ORF between 147 and 1635 nucleotides (496 aa), for M-G1 segment 1931 bp ORF between 3200 and 5131 nucleotides (643 aa), and for M-G2 segment 1564 bp ORF between 1637 and 3200 nucleotides (521 aa) were isolated. For L segment, the 11,835 bp ORF between 78 and 11,912 nucleotides were divided into nine segments composed of six fragments with a size of 1500 bp (500 aa), one fragment with a size of 1506 bp (502 aa) and one fragment with a size of 1029 bp (343 aa). Among these nine segments, 130 bp fragments overlapped with each other (Supplementary Table S1). Homologous recombination took place between the PCR product and linearized pXT7 vector in competent DH5a cells. The recombinant plasmids were then isolated from this culture using QIAprep Mini Kit (Qiagen, USA) according to the manufacturer’s protocol. The purified plasmids expressing recombinant proteins with hemagglutinin (HA) tag at 3′ end and a polyhistidine (His) tag at the 5′ end were visualized by agarose gel electrophoresis (Supplementary Fig. S6) and sequenced.

### Expression levels of CCHFV proteins in *E. coli*

The purified pXT7 plasmids were transformed into *E. coli* BL21 Star (DE3) pLysS chemically competent cells according to the manufacturer’s protocol (Thermoscientific, USA). *E. coli* BL21 Star (DE3) pLysS cells containing 13 different plasmids were incubated with vigorous shaking at 37 °C up to an optical density (OD600) at of 0.4. Then, expression was induced with 0.5 mM IPTG (*isopropyl-D-thiogalactopyranoside*) for 4 h. Next, *E. coli* cells were harvested by centrifugation at 5000×*g* for 10 min. The cell pellets were homogenized with lysis buffer [0.1% Triton X-100, 50 mM Tris–Cl and 0.3 M NaCl (pH 7.4)] followed by 3 × freeze-thawing and then the processed samples were centrifuged at 30,000×*g* for 20 min at 4 °C. After centrifugation, the supernatants were incubated with 1 ml Ni–NTA Superflow beads (Qiagen, USA) shaking for 30 min. Next, the suspension was centrifuged at 2000×g for 1 min and the supernatant was discarded. Thereafter, the Ni–NTA beads containing recombinant proteins were washed with 50 mM Tris–Cl and 0.3 M NaCl and 25 mM imidazole (pH 7.4) for 30 min with shaking to remove the nonspecific bindings and centrifuged at 2000×g for 1 min. Ni–NTA beads containing recombinant proteins were incubated with 50 mM Tris–Cl and 0.3 M NaCl and 0.5 M imidazole (pH 7.4) for 30 min with shaking to release the proteins and centrifuged at 2000×g for 1 min. The molecular weight and level of recombinant protein expression in supernatants was determined by Western blotting as described below. The most abundantly expressed recombinant proteins were selected to be produced in larger amount and used as antigen in Rec-ELISA.

### Protein expression and purification of selected antigens

According to the protein expression levels, two proteins of CCHF (Nucleocapsid and Mucin-like variable domain) were selected to be used as antigen in Rec-ELISA. Protein expression and purification of selected antigens was performed as previously described^44–47^. Initially, *E. coli* BL21 (DE3) cells (Thermoscientific, USA) were transformed with pXT7 vectors encoding full length CCHFV Kelkit Strain S-segment (named pXT7/rNP) and CCHFV Kelkit strain M-segment Mucin like variable domain (named pXT7/rMLD). *E. coli* BL21 cells with pXT7/rNP and pXT7/MLD plasmids were grown using bioreactor (Bioflo 110, New Brunswick, USA). Initially, 500 ml LB medium containing above mentioned plasmids was incubated overnight and transferred into the bioreactor containing 7.5 L enrichment medium and vigorously shake at 400 rpm and 37 °C. The dissolved oxygen level was maintained at 40–60 (ppm) and pH was kept at 7.0 ± 0.4. As the OD600 reached 0.4, cells were induced with 0.5 mM IPTG and incubated at 37 °C for 4 h.

At the end of upstream processing, the cells were pelleted by centrifugation at 5000×*g*. Next, the pellet was homogenized with ice cold loading buffer (50 mM Tris–Cl, pH 7.5, 0.3 M NaCl) using a blender (Waring, USA). Then, the cells were disrupted using microfluidizer processor (M-110L, Microfluidics, USA) using an internal pressure of 18,000 psi. Thereafter, the disrupted sample was centrifuged at 30,000×*g* for 30 min at 4 °C and clarified supernatant was immediately passed through a 0.45 mm filter (Corning, USA). To purify the recombinant proteins from filtered supernatant, a liquid chromatography system which is controlled by UNICORN Version 5.11 software (URL: https://www.cytivalifesciences.com/) was used (AKTA FPLC, Cytiva, USA). During purification, filtered supernatant was applied to a 5 ml HiTrap Chelating HP column (Cytiva, USA) and the column was washed initially with 150 mM imidazole containing 50 mM Tris–Cl, pH 7.5, 0.3 M NaCl buffer to remove junk protein. Recombinant proteins were released from column by elution buffer containing 300 mM imidazole. Recombinant proteins were detected by UV280 and shown on 12% sodium dodecyl sulfate polyacrylamide gel (SDS-PAGE). The fractions containing recombinant protein were concentrated with a Vivaspin filter unit (Sartorius, Germany) at 4 °C. Polishing was achieved with a Superdex 200 gel filtration column (Cytiva, USA) and recombinant proteins were detected by UV280 and quantitated by Bradford method using Comassie blue protein assay reagent and serially diluted serum bovine albumin (Pierce, USA). The resulting recombinant proteins were named as rNP and rMLD.

### SDS-PAGE and Western blotting

SDS-PAGE and Western blot to show the recombinant proteins was performed as previously described^44–47^. Separation of recombinant proteins was achieved by 12% SDS-PAGE. Then, the separated proteins were transferred to a polyvinylidene difluoride (PVDF) transfer membrane (Immobilon-P, Millipore, USA). The membranes were blocked with 6.25% non-fat dry milk containing 1xTBS-T buffer (20 mM Tris–Cl pH: 7.8, 0.5 M NaCl, 0.5% Tween 20). Next, the membranes were probed with monoclonal anti-polyhistidine antibody (1:2000) (Sigma, USA) in 1xTBS-T for 1.5 h. Thereafter, the membranes were washed three times with 1xTBS-T and probed with a alkaline phosphatase-conjugated goat anti-mouse IgG (H + L) antibody (1:2500) (Sigma, USA) in 1xTBS-T for 1 h. After incubation, the membranes were washed three times with 1xTBS-T and subsequently with 1xTBS. The blots were developed with diethanolamine buffer (10% Diethanolamine, 0.5 mM MgCl_2_-6H_2_O, pH: 9.8) containing 4.3% 5-bromo-4-chloro-3- indolyl phosphate (diluted in dimethylacetamide) and 4.1% Nitro-BT (diluted in 70% (v/v) dimethylformamide) (Applichem, Germany).

### Recombinant ELISA

Recombinant ELISA (Rec-ELISA) was performed as described previously^34,45,46^ with modifications. Briefly, microtiter plates (Nunc, USA) were washed three times with 300 µl PBS-T [PBS (pH 7.3) containing 0.05% (v/v) Tween 20] and coated overnight at 4 °C with 100 μl of purified rNP and rMLD individually or combination of rNP and rMLD (rNP/rMLD) diluted in PBS at a concentration of 5 µg/ml for each protein. To determine the concentration of recombinant proteins to be used as antigen, serial dilutions of antigen (1.25–2.5–5 and 10 µg/ml) were tested with ELISA using sera of CCHFV confirmed cases. Next day, plates were washed thrice with PBS-T and blocked with 5% nonfat dry milk prepared in 0.05% PBS-T for 30 min. Meanwhile, human sera diluted to 1/100 in blocking buffer supplemented with *E. coli* lysate at a final concentration of 10 mg/ml protein to block anti-*E. coli* antibodies were pre-incubated for 30 min. The non-transformed *E. coli* BL21 (DE3) cells (Thermoscientific, USA) were grown overnight in 250 ml LB with vigorous shaking at 37 °C and pelleted at 5000×*g* for 10 min. Next, the supernatant is discarded, and the weighted pellet is resuspended in 1xPBS-T buffer to reach a final concentration of 100 mg/ml and passed through microfluidizer twice. The processed suspension is kept at − 80 °C freezer.

Thereafter, each well of the plates were probed with blocked human sera in duplicate for 2 h at 37 °C. After incubation, the plates were washed thrice with PBS-T and probed with peroxidase conjugated anti-human IgG diluted in PBS-T at a concentration of 1:5000 (Sigma, USA) for 1 h at 37 °C. Next, plates were washed thrice with PBS-T and bound antibodies were visualized after adding 3,3′,5,5′ tetramethylbenzidine (TMB) substrate. Reaction was stopped by adding 75 ml of 2 N sulfuric acid and the results were evaluated in a micro titer plate reader (Bio-Tek ELx808, USA) at 450 nm. Samples were considered positive if the absorbance value (AV) of the CCHF serum sample exceeded the mean AV + 2 S.D. of the control serum samples (= cut-off level). Control sera samples were collected from healthy blood donors. Each plate contained a set of negative control sera (n:8) selected from the control sera (n: 43) (please see the Supplementary Table S2 for the specific absorbance values of control sera). Each plate contained anti-polyhistidine antibody (1:2500) (Sigma, USA) and alkaline phosphatase-conjugated goat anti-mouse IgG (H + L) antibody as secondary antibody (1: 2500 (Sigma, USA) probed control wells to determine the presence of His tagged proteins.

### Statistical analyses and graphing

Data of age (*y*) and gender and absorbance values as an optical density (OD) obtained from the Rec-ELISA for both CCHF patients and control subjects were analyzed with GraphPad Prism Version 6.0 (GraphPad Software, San Diego, CA, USA; URL: https://www.graphpad.com/). Informational data were retrieved from the electronic patient charts. Descriptive statistics were presented as frequencies, percentages for categorical variables and as mean ± standard deviation (range) and median [interquartile range (IQR)] for continuous variables. Continuous variables were tested firstly for normal distribution by one-sample Kolmogorov–Smirnov and Shapiro–Wilk tests. In comparing the groups, the *Student’s t* test was used for continuous variables and the Chi-square and Fisher’s exact tests were used for categorical variables. Data for the consecutive paired samples for the acute and convalescent phases were compared by the Wilcoxon signed-rank test. The sensitivity, specificity, positive predictive value (PPV), negative predictive value (NPV) and likelihood ratio of the diagnostic tests were calculated by GraphPad Prism. All tests were two-tailed and *P* < 0.05 was considered as significant. Charts were plotted using GraphPad Prism. The correlation between Rec-ELISAs using rNP, rMLD, and rNP/rMLD were evaluated by Pearson correlation and represented with r^2^ value as calculated by Excel programme of Microsoft Office Version 365 (Microsoft 365, USA; URL: https://www.office.com/).

### Ethics statement

The study was approved by the Local Research Ethics Committee of the Ege University, Faculty of Medicine (Protocol # 13-1/6). Written informed consent was provided to all patients and control group of individuals. Written informed consent from parents/legal guardian of minor participants and next of kin and/or legal guardian of deceased participants are obtained for the study purpose. Data privacy protection was guaranteed by anonymization of serum samples.

## Supplementary Information

Supplementary Information

## Data Availability

The datasets used and analyzed during the current study are available from the corresponding author on reasonable request.

## References

[1] Elaldi N, Kaya S (2014). Crimean-Congo Hemorrhagic fever. J. Microbiol. Infect. Dis..

[2] Bente DA (2013). Crimean-Congo hemorrhagic fever: History, epidemiology, pathogenesis, clinical syndrome an genetic diversity. Antiviral. Res..

[3] Leblebicioglu H (2016). Healthcare-associated Crimean-Congo haemorrhagic fever in Turkey, 2002–2014: A multicentre retrospective cross-sectional study. Clin. Microbiol. Infect..

[4] Gruber CEM (2019). Geographical variability affects CCHFV detection by RT-PCR: A tool for in-silico evaluation of molecular assays. Viruses.

[5] Schuster I (2016). A competitive ELISA for species-independent detection of Crimean-Congo hemorrhagic fever virus specific antibodies. Antiviral. Res..

[6] Casals J (1969). Antigenic similarity between the virus causing Crimean hemorrhagic fever and Congo virus. Proc. Soc. Exp. Biol. Med..

[7] Yilmaz GR (2009). The epidemiology of Crimean-Congo hemorrhagic fever in Turkey, 2002–2007. Int. J. Infect. Dis..

[8] Gunes T (2009). Crimean-Congo hemorrhagic fever virus in high-risk population, Turkey. Emerg. Infect. Dis..

[9] Ertugrul B (2009). An outbreak of Crimean-Congo hemorrhagic fever in western Anatolia, Turkey. Int. J. Infect. Dis..

[10] Walter CT, Barr JN (2011). Recent advances in the molecular and cellular biology of bunyaviruses. Gen. Virol..

[11] Ergönül O (2006). Crimean-Congo haemorrhagic fever. Lancet Infect. Dis..

[12] Dowall SD, Richards KS, Graham VA, Chamberlain J, Hewson R (2012). Development of an indirect ELISA method for the parallel measurement of IgG and IgM antibodies against Crimean-Congo haemorrhagic fever (CCHF) virus using recombinant nucleoprotein as antigen. J. Virol. Methods..

[13] Liu D (2014). Fine epitope mapping of the central immunodominant region of nucleoprotein from Crimean-Congo hemorrhagic fever virus (CCHFV). PLoS One.

[14] Saijo M (2002). Recombinant nucleoprotein-based enzyme-linked immunosorbent assay for detection of immunoglobulin G antibodies to Crimean-Congo hemorrhagic fever virus. J. Clin. Microbiol..

[15] Saijo M (2005). Recombinant nucleoprotein-based serological diagnosis of Crimean-Congo hemorrhagic fever virus infections. J. Med. Virol..

[16] Samudzi RR, Leman PA, Paweska JT, Swanepoel R, Burt FJ (2012). Bacterial expression of Crimean-Congo hemorrhagic fever virus nucleoprotein and its evaluation as a diagnostic reagent in an indirect ELISA. J. Virol. Methods.

[17] Rangunwala A, Samudzi RR, Burt FJ (2014). Detection of IgG antibody against Crimean-Congo haemorrhagic fever virus using ELISA with recombinant nucleoprotein antigens from genetically diverse strains. Epidemiol. Infect..

[18] Sas MA (2018). A novel double-antigen sandwich ELISA for the species-independent detection of Crimean-Congo hemorrhagic fever virus-specific antibodies. Antiviral Res..

[19] Emmerich P (2018). Sensitive and specific detection of Crimean-Congo Hemorrhagic Fever Virus (CCHFV)-Specific IgM and IgG antibodies in human sera using recombinant CCHFV nucleoprotein as antigen in μ-capture and IgG immune complex (IC) ELISA tests. PLoS Negl. Trop. Dis..

[20] Shrivastava N (2019). Development of multispecies recombinant nucleoprotein-based indirect ELISA for high-throughput screening of Crimean-Congo hemorrhagic fever virus-specific antibodies. Front. Microbiol..

[21] Atkinson R, Burt F, Rybicki EP, Meyers AE (2016). Plant-produced Crimean-Congo haemorrhagic fever virus nucleoprotein for use in indirect ELISA. J. Virol. Methods.

[22] Levingston Macleod JM, Marmor H, García-Sastre A, Frias-Staheli N (2015). Mapping of the interaction domains of the Crimean-Congo hemorrhagic fever virus nucleocapsid protein. J. Gen. Virol..

[23] Sanchez AJ, Vincent MJ, Nichol ST (2002). Characterization of the glycoproteins of Crimean-Congo hemorrhagic fever virus. J. Virol..

[24] Sanchez AJ, Vincent MJ, Erickson BR, Nichol ST (2006). Crimean-congo hemorrhagic fever virus glycoprotein precursor is cleaved by Furin-like and SKI-1 proteases to generate a novel 38-kilodalton glycoprotein. J. Virol..

[25] Bergeron É (2015). Recovery of recombinant Crimean Congo hemorrhagic fever virus reveals a function for non-structural glycoproteins cleavage by furin. PLoS Pathog..

[26] Bergeron E, Albariño CG, Khristova ML, Nichol ST (2010). Crimean-Congo hemorrhagic fever virus-encoded ovarian tumor protease activity is dispensable for virus RNA polymerase function. J. Virol..

[27] Bartolini B (2019). Laboratory management of Crimean-Congo haemorrhagic fever virus infections: Perspectives from two European networks. Euro Surveill..

[28] WHO. WHO R&D Blueprint: Priority Diagnostics for CCHF Use Scenarios and Target Product Profiles. http://www.searo.who.int/docs/default-source/blue-print/call-for-comments/who-cchf-tpp-dx-draft-v1-0.pdf?sfvrsn=a5b8580_2 (2019).

[29] Anonymous. Crimean-Congo Hemorrhagic Fever (CCHF). Center for Disease Control and Prevention (CDC). https://www.cdc.gov/vhf/crimean-congo/diagnosis/index.html (2020).

[30] Kaya S (2014). Sequential determination of serum viral titers, virus-specific IgG antibodies, and TNF-α, IL-6, IL-10, and IFN-γ levels in patients with Crimean-Congo hemorrhagic fever. BMC Infect. Dis..

[31] Duh D (2007). Viral load as predictor of Crimean-Congo hemorrhagic fever outcome. Emerg. Infect. Dis..

[32] Mazzola LT, Kelly-Cirino C (2019). Diagnostic tests for Crimean-Congo haemorrhagic fever: A widespread tickborne disease. BMJ Glob. Health.

[33] Weidmann M (2016). Biosafety standards for working with Crimean-Congo hemorrhagic fever virus. J. Gen. Virol..

[34] Aubert D (2000). Recombinant antigens to detect Toxoplasma gondii-specific immunoglobulin G and immunoglobulin M in human sera by enzyme immunoassay. J. Clin. Microbiol..

[35] Burt FJ, Leman PA, Abbott JC, Swanepoel R (1994). Serodiagnosis of Crimean-Congo haemorrhagic fever. Epidemiol. Infect..

[36] Ergunay K (2014). Antibody responses and viral load in patients with Crimean-Congo hemorrhagic fever: A comprehensive analysis during the early stages of the infection. Diagn. Microbiol. Infect. Dis..

[37] Bodur H, Akinci E, Ascioglu S, Öngürü P, Uyar Y (2012). Subclinical infections with Crimean-Congo hemorrhagic fever virus, Turkey. Emerg. Infect. Dis..

[38] Marriott AC, Polyzoni T, Antoniadis A, Nuttall PA (1994). Detection of human antibodies to Crimean-Congo haemorrhagic fever virus using expressed viral nucleocapsid protein. J. Gen. Virol..

[39] Álvarez-Rodríguez B (2020). Characterization and applications of a Crimean-Congo hemorrhagic fever virus nucleoprotein-specific Affimer: Inhibitory effects in viral replication and development of colorimetric diagnostic tests. PLoS Negl. Trop. Dis..

[40] Siegel JD, Rhinehart E, Jackson M, Chiarello L, Health Care Infection Control Practices Advisory Committee (2007). 2007 guideline for isolation precautions: Preventing transmission of infectious agents in health care settings. Am. J. Infect. Control..

[41] Leblebicioglu H (2012). Case management and supportive treatment for patients with Crimean-Congo Hemorrhagic fever. Vector Borne Zoonot. Dis..

[42] Davies DH (2005). Profiling the humoral immune response to infection by using proteome microarrays: High-throughput vaccine and diagnostic antigen discovery. Proc. Natl. Acad. Sci. USA.

[43] Döşkaya M (2018). Discovery of new Toxoplasma gondii antigenic proteins using a high throughput protein microarray approach screening sera of murine model infected orally with oocysts and tissue cysts. Parasit. Vectors.

[44] Döşkaya M (2007). GRA1 protein vaccine confers better immune response compared to codon-optimized GRA1 DNA vaccine. Vaccine.

[45] Döşkaya M (2014). Diagnostic value of a Rec-ELISA using Toxoplasma gondii recombinant SporoSAG, BAG1, and GRA1 proteins in murine models infected orally with tissue cysts and oocysts. PLoS One.

[46] Gedik Y (2016). Immunogenic multistage recombinant protein vaccine confers partial protection against experimental toxoplasmosis mimicking natural infection in murine model. Trials Vaccinol..

[47] Şahar EA (2020). Development of a hexavalent recombinant protein vaccine adjuvanted with Montanide ISA 50 V and determination of its protective efficacy against acute toxoplasmosis. BMC Infect. Dis..

