# Supplementary material for: Development of a novel recombinant ELISA for the detection of Crimean-Congo hemorrhagic fever virus IgG antibodies
Source: Sci Rep. 2021 Mar 15;11:5936. doi: 10.1038/s41598-021-85323-1 (PMC7961021; doi:10.1038/s41598-021-85323-1)
Supplement: Supplementary file 1 — Supplementary Information [file 41598_2021_85323_MOESM1_ESM.docx]

**Supplementary Information**

**Title of the manuscript:** Development of a Novel Recombinant ELISA for the Detection of Crimean-Congo Hemorrhagic Fever Virus IgG Antibodies

**Author List:** Sultan Gülce-İz, Nazif Elaldı, Hüseyin Can, Esra Atalay Şahar, Muhammet Karakavuk, Aytül Gül, Gizem Örs Kumoğlu, Aysu Değirmenci Döşkaya, Adnan Yüksel Gürüz, Aykut Özdarendeli, Philip Louis Felgner, Huw Davies, Mert Döşkaya

**Supplementary table 1.** Primer sets to isolate S, M (1-3) and L (1-9) segments of Turkey-Kelkit06 strain and the theoretical molecular weights of ORFs isolated by these primer sets.

| **GenBank Accession no** | **Gene size (bp)** | **Molecule Location** | **Segment** | **The positions of primers to isolate the ORFs (bp)** | **Amlicon Size (bp)** | **5' primer isolation primer** | **3' primer isolation primer** | **Protein Product** | **ORF Size (aa)** | **Theoretical MW of ORFs (kDa)** |
| --- | --- | --- | --- | --- | --- | --- | --- | --- | --- | --- |
| GQ337053.1 | 1673 | CCHFV-Kelkit-S | S | 56-1503 | 1448 | acgacaagcatatgctcgagATGGAAAACAAGATCGAGGT | tccggaacatcgtatgggTAGATGATGTTGGCACTGGTGG | nucleocapsid protein | 482 | 53.9 |
| GQ337054.1 | 5364 | CCHFV-Kelkit-M-mucin like | M-Mucin like | 147-1635 | 1489 | acgacaagcatatgctcgagGGAGCTCATGGGCAGTTAAA | tccggaacatcgtatgggTAAAGCCTGTTGATGCCTTGC | envelope glycoprotein | 496 | 53.67 |
| GQ337054.1 | 5364 | CCHFV-Kelkit-M-G1 | M-G1 | 3200-5131 | 1931 | acgacaagcatatgctcgagAAACCTCTTTTCTTGGACAG | tccggaacatcgtatgggGTTTTTGTGGAGAACAGCTCAG | envelope glycoprotein | 643 | 72.19 |
| GQ337054.1 | 5364 | CCHFV-Kelkit-M-G2 | M-G2 | 1637-3200 | 1564 | acgacaagcatatgctcgagagGAAACTACTGTCCGAAGA | tccggaacatcgtatgggTCTGAGCACAAGAGCTTCGT | envelope glycoprotein | 521 | 57.99 |
| GQ337055.1 | 12149 | CCHFV-Kelkit-L1 | L | 78-1583 | 1506 | acgacaagcatatgctcgagATGGACTTCTTGAGGAACCT | tccggaacatcgtatgggtaGCGGGTGAGGTCACTTTCAG | polymerase | 502 | 56.85 |
| GQ337055.1 | 12149 | CCHFV-Kelkit-L2 | L | 1433-2933 | 1500 | acgacaagcatatgctcgagAGTATGTCCAAATTGGATAA | tccggaacatcgtatgggtaTAATCCTGTTGGCCTATTGA | polymerase | 500 | 57.2 |
| GQ337055.1 | 12149 | CCHFV-Kelkit-L3 | L | 2783-4283 | 1500 | acgacaagcatatgctcgagACTGACATAGTTGTTGGAGC | tccggaacatcgtatgggtaCCTGTTCAAATAGAAAGGTC | polymerase | 500 | 57.17 |
| GQ337055.1 | 12149 | CCHFV-Kelkit-L4 | L | 4133-5633 | 1500 | acgacaagcatatgctcgagTTTAATAGATCAGGGGTTAA | tccggaacatcgtatgggtaTCTAAGCATCTTAACTATCT | polymerase | 500 | 576.7 |
| GQ337055.1 | 12149 | CCHFV-Kelkit-L5 | L | 5483-6983 | 1500 | acgacaagcatatgctcgagGCAAGGAGAGACCCGAAGAA | tccggaacatcgtatgggtaGTTTCTACTGAACATCTCAG | polymerase | 500 | 57.09 |
| GQ337055.1 | 12149 | CCHFV-Kelkit-L6 | L | 6833-8333 | 1500 | acgacaagcatatgctcgagCAATTAGCTTTTGCACAGGC | tccggaacatcgtatgggtaCAGAGGCCTATTGAGCTCAA | polymerase | 500 | 55.4 |
| GQ337055.1 | 12149 | CCHFV-Kelkit-L7 | L | 8183-9683 | 1500 | acgacaagcatatgctcgagTCAAGCAGCTTGACCACATT | tccggaacatcgtatgggtaCAAGTTACCTTTCAGCATGT | polymerase | 500 | 56.29 |
| GQ337055.1 | 12149 | CCHFV-Kelkit-L8 | L | 9533-11033 | 1500 | acgacaagcatatgctcgagACAGATGAATCATCAGACTC | tccggaacatcgtatgggtaTCTGTCAATGTTAACAAGCA | polymerase | 500 | 56.52 |
| GQ337055.1 | 12149 | CCHFV-Kelkit-L9 | L | 10883-11912 | 1029 | acgacaagcatatgctcgagCTCGAACAAGGTGTAGAAGA | tccggaacatcgtatgggtaATCTGAATCCCAGTTAAAGC | polymerase | 343 | 38.85 |

**Supplementary table 2.** Absorbance values (AV) of Control sera as detected by Rec-ELISA using rMLD/rNP. Samples were considered positive if the mean AV value of the serum sample exceeded the mean AV+2 S.D. of all sera.

| **Sample no** | **AV well 1** | **AV well 2** | **mean OD value** | **Interpretation of the result** |
| --- | --- | --- | --- | --- |
| 1 | 0,044 | 0,063 | 0,054 | Negative |
| 2 | 0,085 | 0,095 | 0,090 | Negative |
| 3 | 0,200 | 0,184 | 0,192 | Negative |
| 4 | 0,149 | 0,154 | 0,152 | Negative |
| 5 | 0,308 | 0,295 | 0,302 | Negative |
| 6 | 0,100 | 0,132 | 0,116 | Negative |
| 7 | 0,060 | 0,120 | 0,090 | Negative |
| 8 | 0,099 | 0,146 | 0,123 | Negative |
| 9 | 0,142 | 0,105 | 0,124 | Negative |
| 10 | 0,101 | 0,089 | 0,095 | Negative |
| 11 | 0,105 | 0,101 | 0,103 | Negative |
| 12 | 0,254 | 0,187 | 0,221 | Negative |
| 13 | 0,130 | 0,046 | 0,088 | Negative |
| 14 | 0,126 | 0,102 | 0,114 | Negative |
| 15 | 0,244 | 0,399 | 0,322 | Negative |
| 16 | 0,067 | 0,107 | 0,087 | Negative |
| 17 | 0,071 | 0,283 | 0,177 | Negative |
| 18 | 0,308 | 0,294 | 0,301 | Negative |
| 19 | 0,164 | 0,197 | 0,181 | Negative |
| 20 | 0,129 | 0,322 | 0,226 | Negative |
| 21 | 0,246 | 0,265 | 0,256 | Negative |
| 22 | 0,131 | 0,139 | 0,135 | Negative |
| 23 | 0,105 | 0,105 | 0,105 | Negative |
| 24 | 0,019 | 0,037 | 0,028 | Negative |
| 25 | 0,091 | 0,150 | 0,121 | Negative |
| 26 | 0,057 | 0,274 | 0,166 | Negative |
| 27 | 0,013 | 0,224 | 0,119 | Negative |
| 28 | 0,074 | 0,348 | 0,211 | Negative |
| 29 | 0,110 | 0,149 | 0,130 | Negative |
| 30 | 0,284 | 0,290 | 0,287 | Negative |
| 31 | 0,141 | 0,116 | 0,129 | Negative |
| 32 | 0,200 | 0,060 | 0,130 | Negative |
| 33 | 0,172 | 0,139 | 0,156 | Negative |
| 34 | 0,076 | 0,083 | 0,080 | Negative |
| 35 | 0,063 | 0,042 | 0,053 | Negative |
| 36 | 0,251 | 0,249 | 0,250 | Negative |
| 37 | 0,304 | 0,228 | 0,266 | Negative |
| 38 | 0,512 | 0,447 | 0,480 | **Positive** |
| 39 | 0,008 | 0,006 | 0,007 | Negative |
| 40 | 0,114 | 0,057 | 0,086 | Negative |
| 41 | 0,185 | 0,220 | 0,203 | Negative |
| 42 | 0,192 | 0,276 | 0,234 | Negative |
| 43 | 0,031 | 0,067 | 0,049 | Negative |
|  |  | Mean value of AVs | **0,159** |  |
|  |  | Standard deviation (S.D.) | **0,092** |  |
|  |  | Cut-off value | **0,343** |  |


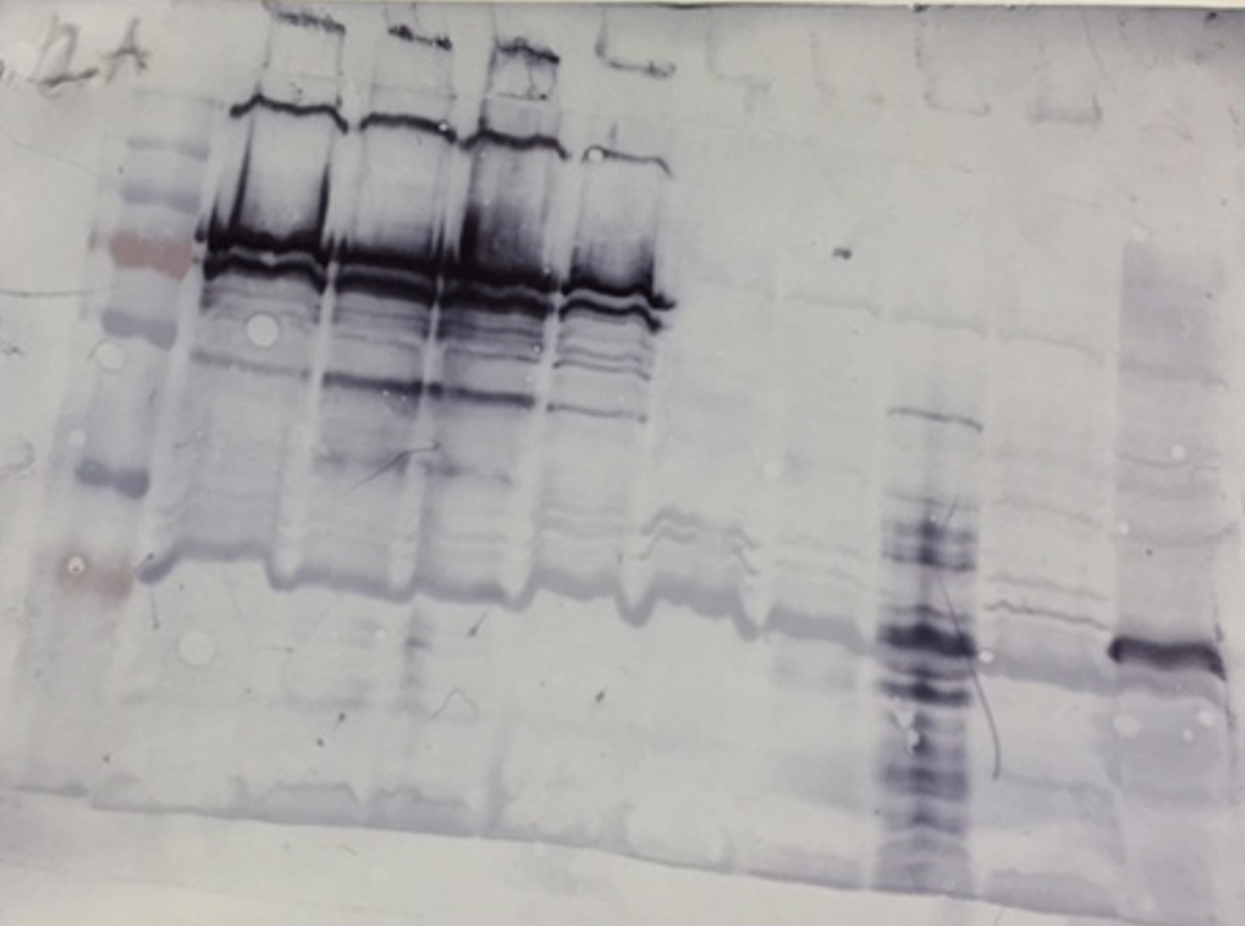


1 2 3 4 5 6 7 8 9 10

**Supplementary Fig. 1.** Protein expression levels of recombinant KS, MLD, G1, G2, L1 and L2 CCHFV proteins as detected by Western blot using anti-polyhistidine antibody. **Lane 1:** Ladder, **Lane 2:** rKS, **Lanes 3-4-5:** rMLD, **Lane 6:** G1, **Lane 7:** G2, **Lane 8:** L1, **Lane 9:** L2, **Lane 10:** Control protein (recombinant GRA1 protein of *Toxoplasma gondii*).

1 2 3 4 5 6 7 8 9 10


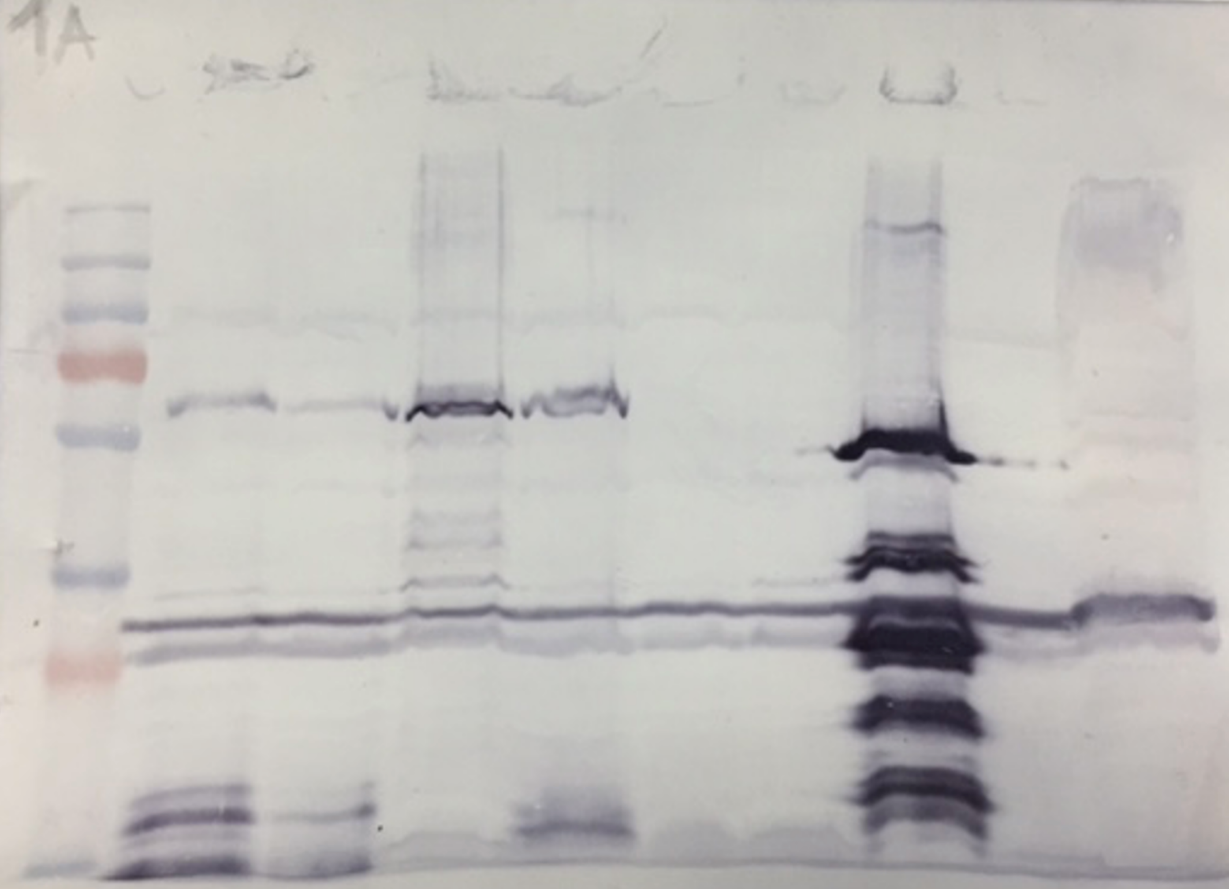


**Supplementary Fig. 2.** Protein expression levels of recombinant L3 through L9 CCHFV proteins as detected by Western blot using anti-polyhistidine antibody. **Lane 1:** Ladder, **Lane 2:** L3, **Lane 3:** L4, **Lane 4:** L5, **Lane 5:** L6, **Lane 6:** L7, **Lane 7:** L8, **Lane 8:** L9, Lane 9 and 10: Control protein (recombinant GRA1 protein of *Toxoplasma gondii*).

1 2 3 4 5 6 7 8 9 10


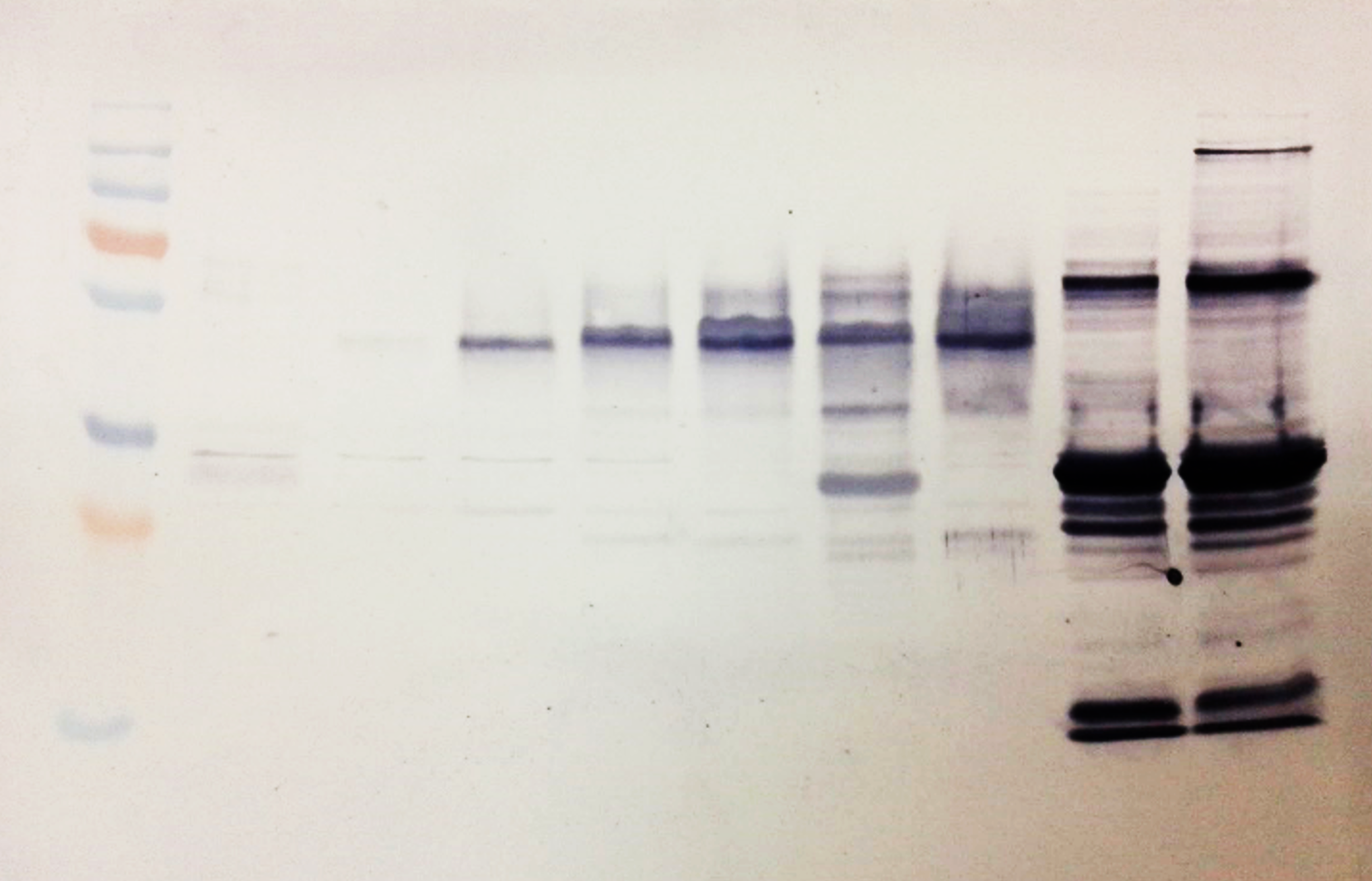


**Supplementary Fig. 3.** Original Western blotting image showing the purified rNP protein shown in Figure 1 of the manuscript. **Lane 4:** rNP protein with a theoretical molecular weight of ~53.9 kDa. The lane 4 on this image is the original Western blot image represented on Figure 1 Lane1. Lane 1: Ladder, Lanes 2-3-5-6-7-8: rNP samples obtained during the chromatography purification. Lanes 9-10: Control protein (recombinant BAG1 protein of *Toxoplasma gondii*)

1 2 3 4 5 6 7 8 9 10


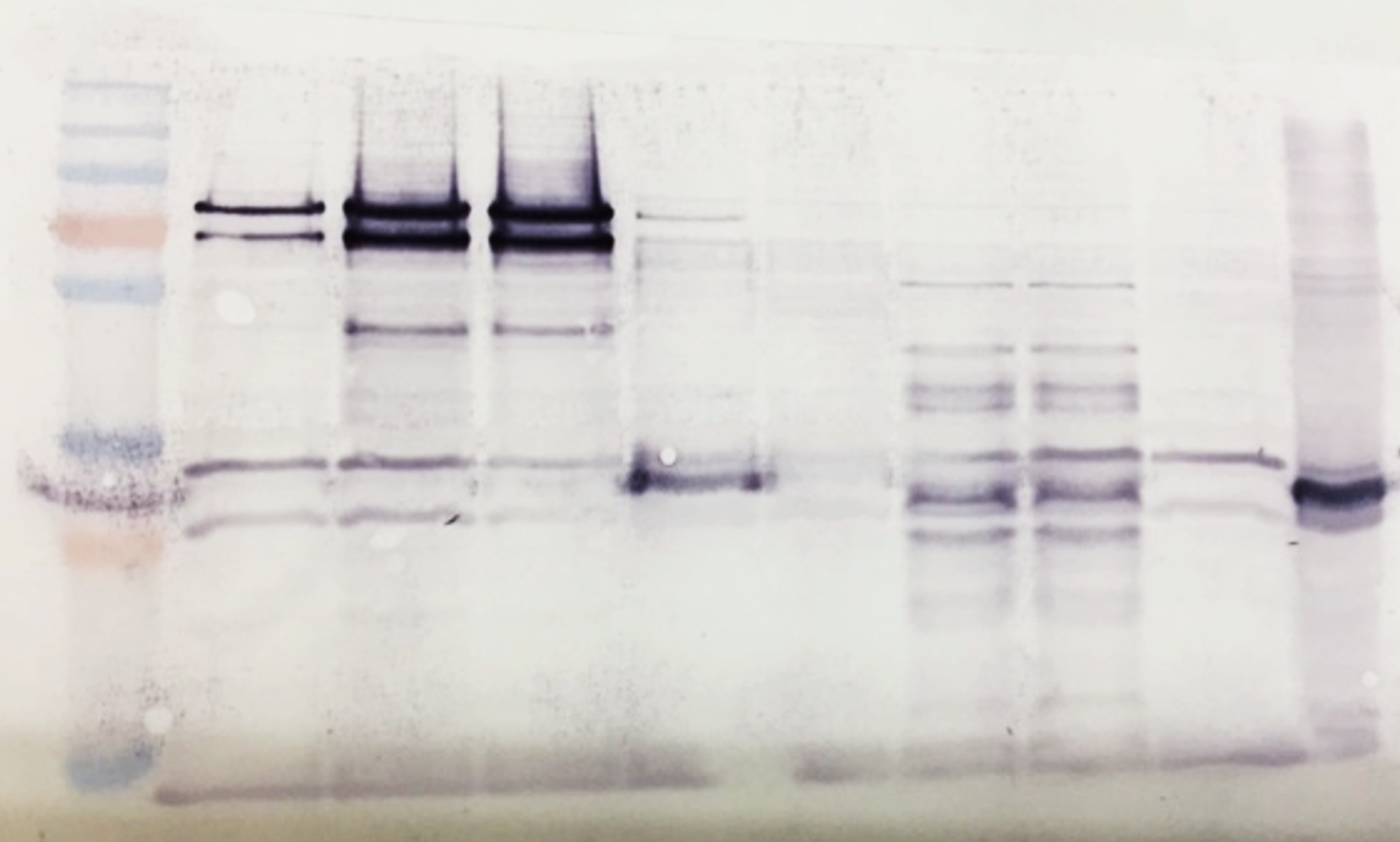


**Supplementary Fig. 4.** Original Western blotting image showing the purified rMLD protein shown in Figure 1 of the manuscript. **Lane 3:** rMLD protein with a theoretical molecular weight of ~53.67 kDa. The lane 3 on this image is the original Western blot image represented on Figure 1 Lane2. Lane 1: Ladder, Lanes 2-3-4: Different samples of rMLD obtained during chromatography purification, Lanes 5-6-7-8-9: different samples obtained during chromatography purification, Lane 10: Control protein (recombinant GRA1 protein of *Toxoplasma gondii*)


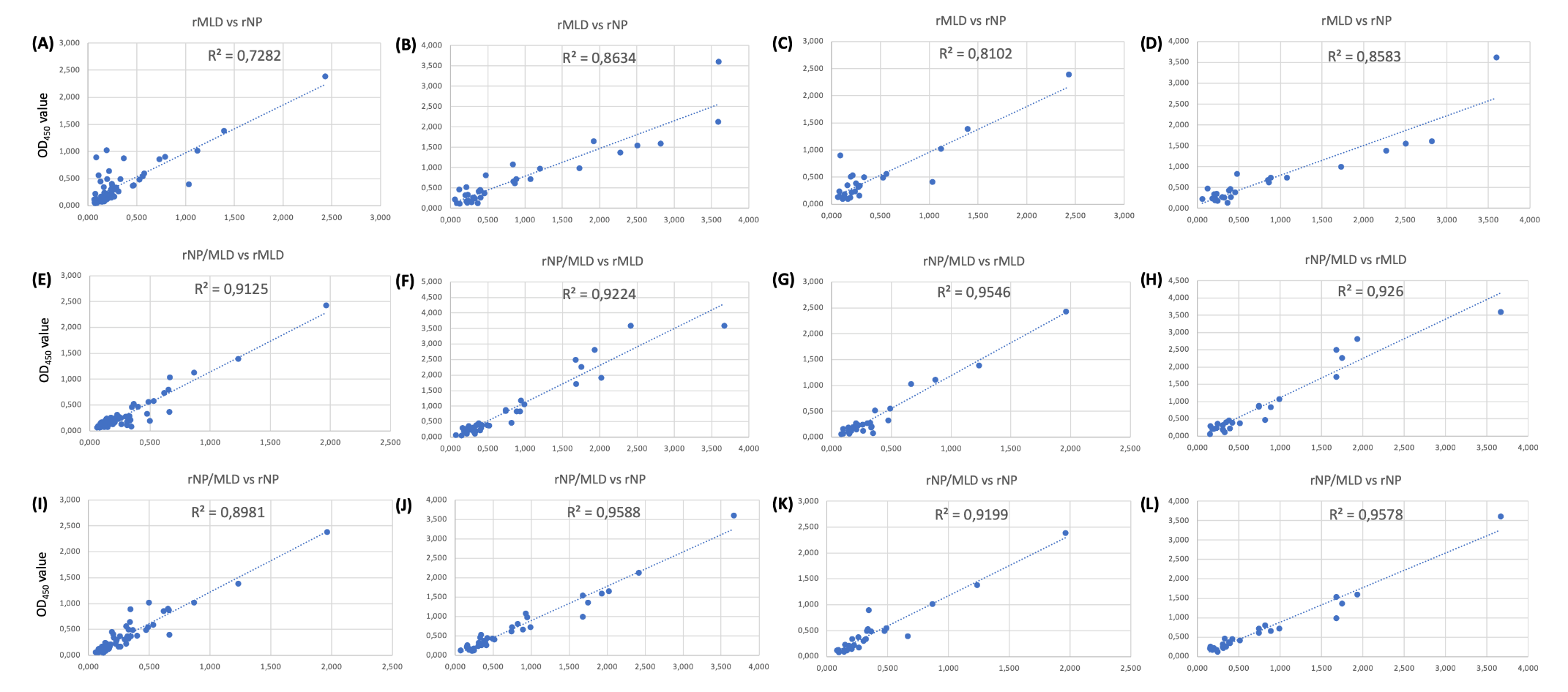


**Supplementary Fig. 5.** Comparison of the Rec-ELISAs using rMLD, rNP and rMLD/rNP using Pearson correlation analyses. **(A-E-I)** Results of acute phase CCHF patients (n: 64); **(B-F-J)** Results of convalescent phase CCHF patients (n: 35); **(C-G-K)** Results of acute phase sera in the group of 25 CCHF patients who had paired serum samples; **(D-H-L)** Convalescent phase sera in the group of 25 CCHF patients who had paired serum samples**.**


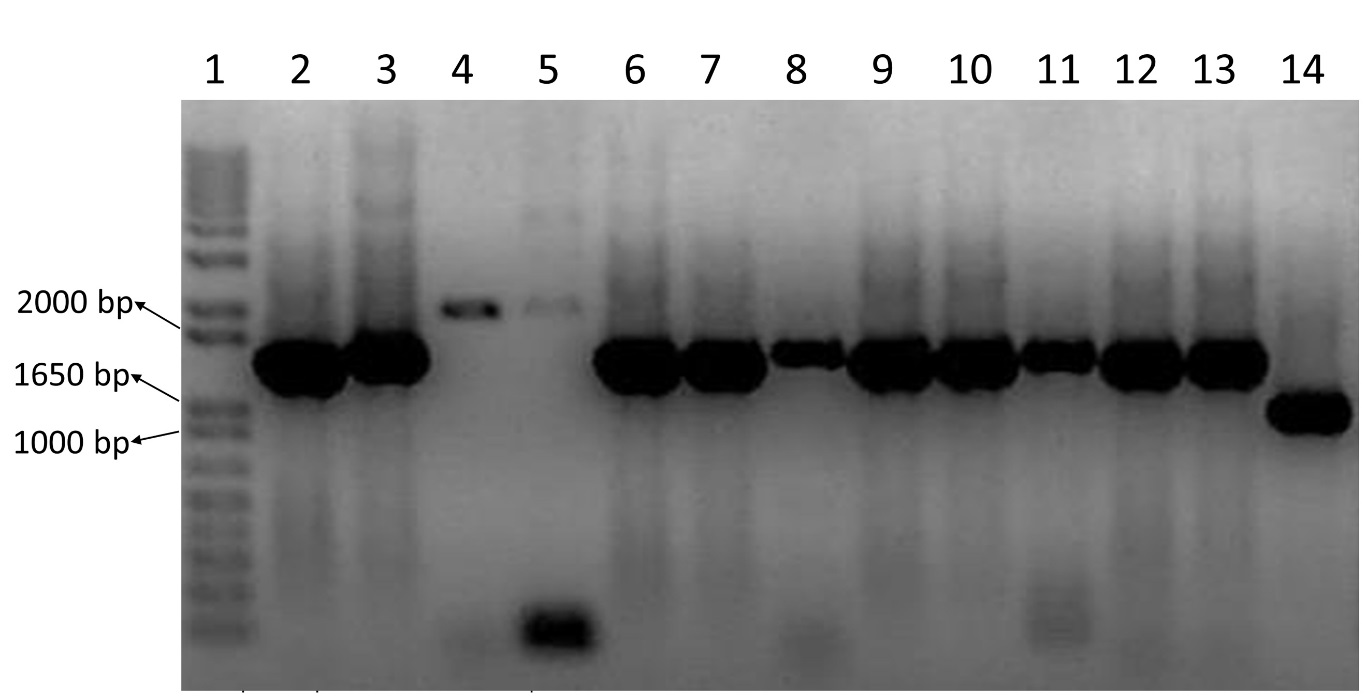


**Supplementary Fig. 6.** PCR amplification of S, M (M1-M3), and L (L1-L9) segments of CCHFV. The PCR products are shown on a single agarose gel image. Lane 1: Ladder, Lane 2: S Segment, Nucleocapsid protein; Lanes 3-5: M-mucin like domain, G1 and G2 segments of Envelope Glycoprotein; Lanes 6-14: L1 through L9 segments of RNA Polymerase.

1 2 3 4 5 6 7 8 9 10 11 12 13 14


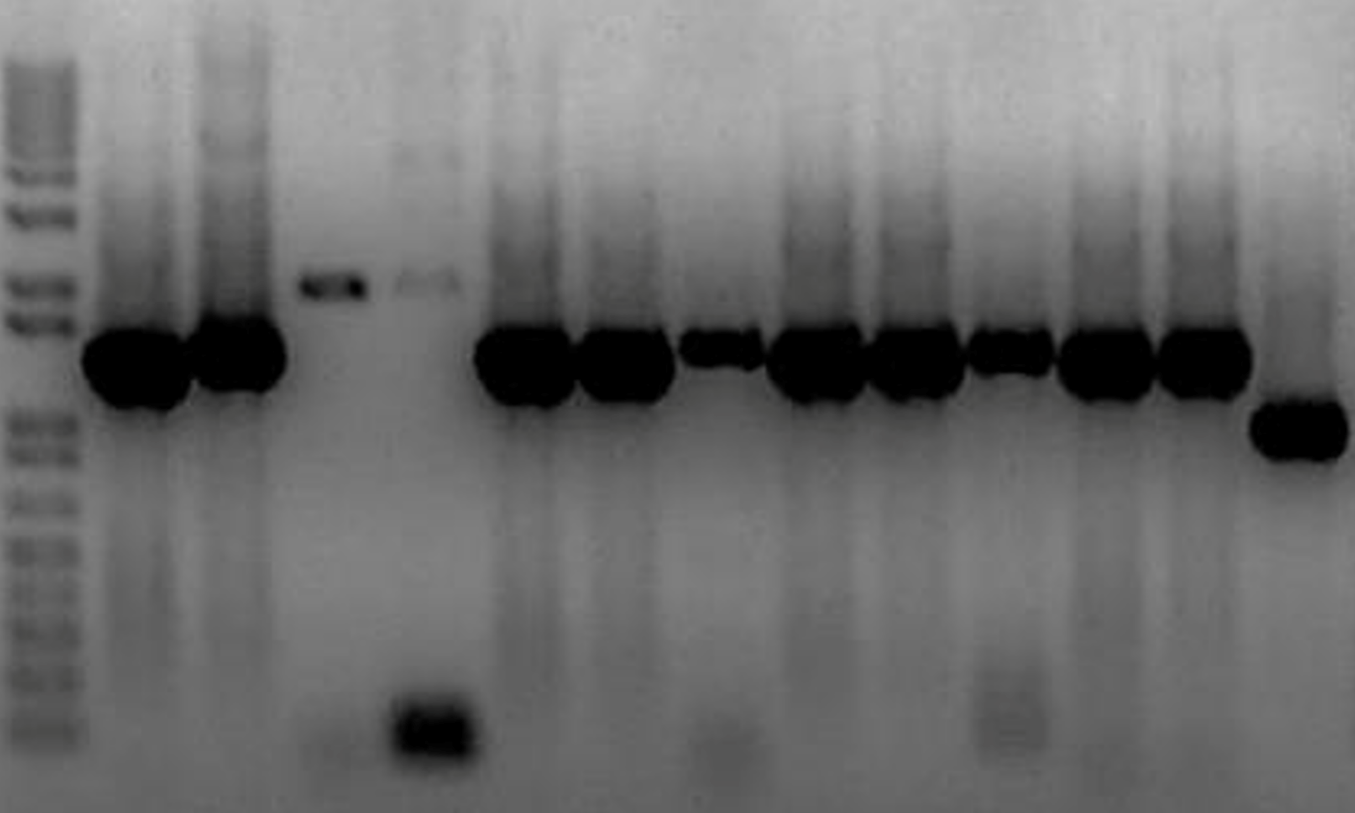


**Supplementary Fig. 7.** Original agarose gel image of Supplementary Fig. 6. PCR amplification of S, M (M1-M3), and L (L1-L9) segments of CCHFV. The PCR products are shown on a single agarose gel image. Lane 1: Ladder, Lane 2: S Segment, Nucleocapsid protein; Lanes 3-5: M-mucin like domain, G1 and G2 segments of Envelope Glycoprotein; Lanes 6-14: L1 through L9 segments of RNA Polymerase.

1 2 3 4 5 6 7 8 9 10


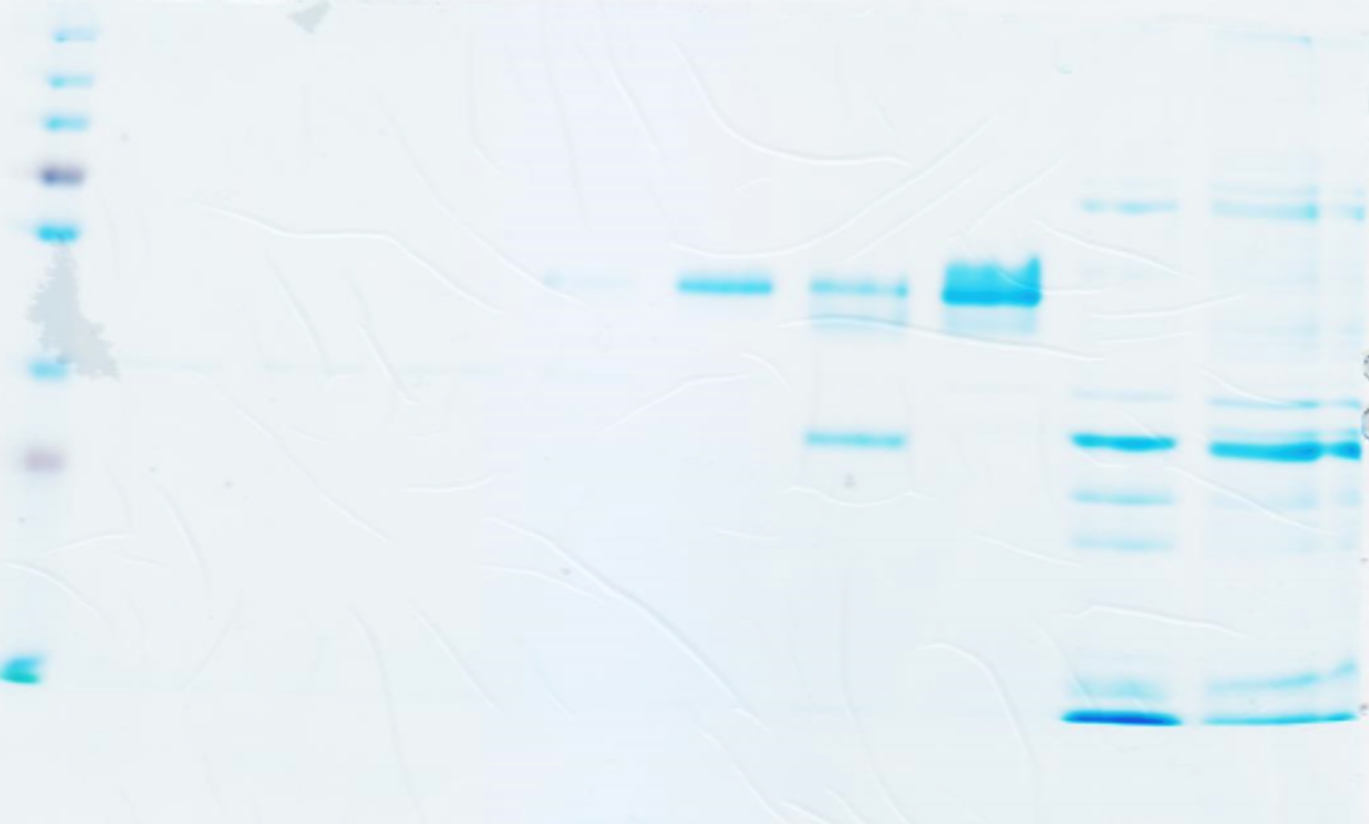


**Supplementary Fig. 8.** SDS-PAGE gel image of rNP protein with a theoretical molecular weight of ~53.9 kDa (stained with commassie blue). **Lane 1:** Ladder, **Lanes 2-8:** rNP samples obtained during the chromatography purification. **Lanes 9-10:** Control protein (recombinant BAG1 protein of *Toxoplasma gondii*)
